# Metal- and covalent-organic frameworks as solid-state electrolytes for metal-ion batteries

**DOI:** 10.1098/rsta.2018.0225

**Published:** 2019-05-27

**Authors:** Elise M. Miner, Mircea Dincă

**Affiliations:** Department of Chemistry, Massachusetts Institute of Technology, Cambridge, MA, USA

**Keywords:** MOF, COF, metal-ion batteries, solid-state electrolyte

## 1. Introduction

Society's long-standing energy demands have fuelled for centuries the quest for power-dense, portable and economically viable energy carriers. Since the birth of the first rechargeable battery in 1860 [1], emerging battery technologies have provided both answers to these demands as well as additional obstacles. One ubiquitous energy storage device, the metal or metal-ion battery, offers quintessential examples of both. The strongly reducing nature of Group 1 and 2 metal ions qualifies these elements as viable energy-dense anode materials: standard reduction potentials several volts below that of the standard hydrogen electrode (SHE) allow a thermodynamically favourable oxidation of these metals to readily release electrons that shuttle through an external circuit, generating the electric current that serves as the power supply during battery discharge. Integration of energy-dense materials into devices allows power sources to be compact and portable, by maximizing energy output per unit mass of material. Further, the reversibility of these oxidation events makes possible extensive battery cycling, thus providing a rechargeable power source. Indeed, current Li-ion batteries boast an energy density of 265 Wh kg^−1^, with the potential of a 20% improvement, and are operable for over 1000 charge–discharge cycles [2].

Although the chemical properties of metal-ion batteries offer impressive performance and exciting possibilities, harnessing the power of such reactive workhorses in a controlled manner comes with its own challenges. In Li-ion batteries, formation of Li dendrites during charging can puncture the battery membrane separating the anode from the cathode, causing a leakage of flammable electrolyte if the electrolyte is liquid, or causing a short-circuit should the dendrites reach the cathode. Another limitation of Li-ion batteries stems from the first charging cycle, which causes the formation of a stable reduction product known as the solid–electrolyte interphase (SEI) layer at the anode due to the fact that the anode and cathode lithiate at potentials outside the stability window of common liquid electrolytes. Formation of the SEI layer diminishes the cathode capacity, thus necessitating a larger amount of cathode material to be incorporated into the battery relative to the anode mass. This additional material lowers the battery's energy density. Another downfall of the charge–discharge process in Li-ion batteries is that a poorly formed SEI will limit battery cycle life due to continuous reactivity of the electrolyte at the anode and irreversible loss of Li^+^ ions [2].

From an economic standpoint, the increasingly widespread adoption of rechargeable batteries also highlights the difference in cost and geopolitical availability between Li metal and more abundant metals such as Na, Mg, K, Ca or Al. These heavier metals are indeed the focus of intense research in the context of electrical energy storage, but present their own challenges. Na, an attractive candidate due to its high abundance, relatively small ionic radius, high specific capacity and low reduction potential (2.71 V versus SHE), has shown more problematic reactivity with organic liquid electrolytes compared to Li and presents the same dendrite formation challenges as Li batteries [3,4]. Mg, another viable candidate, is 5 orders of magnitude more abundant than Li, does not form dendrites during charging, and offers almost double the volumetric capacity of Li (3833 mAh ml^−1^ versus 2062 mAh ml^−1^ for Mg/Mg^2+^ versus Li/Li^+^, respectively). However, the most common commercial electrolytes used in Li-ion batteries are not appropriate choice for Mg-ion batteries because the SEI layer formed in the latter is completely insulating for Mg^2+^, an obvious problem for battery cyclability [2]. Owing to its higher atomic weight, which inherently leads to lower energy density, K has received comparatively less attention than Li and Na as a battery material. However, its abundance and lower cost may offset this handicap, especially considering that K also does not alloy with Al, a popular and cheap current collector that otherwise needs further processing when used in Li-ion batteries. Additionally, the weaker Lewis acidity of K^+^ ions relative to Li^+^ and Na^+^ ions accounts for lower desolvation energy and enhanced transport kinetics across the electrolyte/electrode interface, which ultimately increases ionic conductivity [5]. Lastly, Ca^2+^ features a small ionic radius and a stable divalent oxidation state that would afford higher energy density. It has high natural abundance, and a standard reduction potential close to that of Li^+^ which would allow a high potential window for electrolytes. However, one significant obstacle preventing development of Ca batteries with organic liquid electrolytes is that diffusion of Ca^2+^ ions through the SEI layer prevents re-plating of Ca on the anode during charging [6]. Even further enhancement of capacity can be achieved by taking advantage of trivalent ions such as Al^3+^, which features quadruple the volumetric capacity of Li^+^ (8046 mAh cm^−3^). Although this is a promising feature for energy storage advancement, challenges with Al-ion battery systems containing liquid electrolytes stem from the formation of passive oxide films on the electrode surface and/or from anode corrosion [7].

Efforts have also been made to improve metal and metal-ion battery performance by further optimizing cell components beyond the metal anodes, and in particular the electrolyte. The impedance of all metal-ion batteries is likely elevated due to mobile species besides the active metal ions (e.g. charge-balancing anions, solvent molecules, etc.) during cycling [2]. Pursuing various formulations of anode and cathode materials [8–11], developing new supporting electrolytes, and new solvent or solvent mixtures [12–14] have all been explored as potential solutions to these challenges. The focus of this review is to present an argument for solid-state, rather than liquid, electrolytes in such batteries and to discuss the potential utility of crystallographically ordered, metal- and covalent-organic frameworks (MOFs and COFs), as solid-state electrolytes. The review specifically covers reported MOFs and COFs as solid-state electrolytes, distils metrics for vetting solid electrolyte candidates, and considers future directions for this field.

## 2. Motivation for and evolution of solid-state battery electrolytes

Motivation for a solid-state electrolyte is several-fold. Firstly, solid-state electrolytes would eliminate the hazard of housing a flammable liquid material inside of the battery, enhancing safety. Secondly, a solid-state electrolyte may allow for immobilization of charge-balancing anions, which would allow maximization of the cation transference number. Thirdly, many liquid electrolytes are not stable in the required potential window imposed by the battery electrodes. Solid electrolytes should aim to address all of these challenges. In addition to minimizing the formation of reactive by-products, a more stable electrolyte may prevent the formation of an SEI layer, consequently improving the energy density of the cell by eliminating the need for excess sacrificial cathode material. Elimination of the SEI layer would also increase the viability of Mg and Ca-ion batteries, the development of which is currently limited by the inability of these ions to travel through the SEI layer during charging. Finally, for metal anode batteries, liquid electrolytes provide no morphological control over anodic plating of the metal during battery charging; a solid electrolyte with sufficient mechanical strength may encourage uniform plating, thereby preventing dendrite formation.

Several classes of materials have been evaluated as potential solid electrolytes for metal or metal-ion batteries, including polymers and composites thereof [15], inorganic solids [15] and, as will be discussed further, MOFs and COFs. Polymer electrolytes can offer enhanced potential stability windows and cation transference numbers compared to liquid electrolytes, due to the immobilized anionic hopping sites along the polymer backbone. However, polymers are ineffective at preventing dendrite growth and typically exhibit ambient temperature conductivity values that are too low for commercial applications (10^−8^–10^−5^ S cm^−1^) [2,3]. Additives such as ceramics or ionic liquids have been doped into polymer matrices to enhance ion mobility, creating more conductive polymer composites. Dopants can increase the electrolyte conductivity by 2–3 orders of magnitude, but optimization of the polymer/dopant blend and obtaining mechanistic understanding of the transport pathways in such hybrids is not trivial [16]. Additionally, dopants in the polymer matrix often compromise the electrode–electrolyte interface, and these dopants can exhibit lower electrochemical or chemical stability and form themselves a resistive layer at the electrode [3]. In terms of ionic conductivity and mechanical robustness, inorganic solid electrolytes are among the most promising solid electrolytes thus far. Li_3_OX-based antiperovskites (X = Cl^−^, Br^−^) exhibit Li^+^ activation energies of 0.18–0.26 eV and conductivities of up to 2 × 10^−3^ S cm^−1^ at 25°C, exceeding the conductivities of polymer electrolytes [17]. However, preparation of Li_3_OX antiperovskites involves thermal treatment that inadvertently removes charge-balancing lithium, resulting in decreased charge carrier density. Additionally, challenges exist regarding yield and phase purity for these materials that contribute to poor interfacial contact between the electrode and electrolyte. This, combined with formation of insulating SEI layers, increases battery resistance [18]. Antiperovskites have also been shown to conduct Na^+^ ions, albeit with modest conductivities of *ca* 10^−5^ S cm^−1^ at 160°C and activation energies of 0.6–0.8 eV [19]. Much higher conductivities are observed in *closo*-borate salts *A*CB_9_H_10_ (*A* = Li^+^ or Na^+^), which boast conductivities of 0.03 S cm^−1^ at temperatures above an ordered–disordered phase transition temperature. Noteworthy activation energies of 0.29 eV (Li^+^) and 0.20 eV (Na^+^) and potential stability windows of approximately 5 V were measured. A logistical barrier with these *closo*-borate materials is that the phase transition is reversible, and thus the material must be kept above 127°C (Li^+^) and 107°C (Na^+^) in order to maintain the conductive properties [20]. Another archetypal Na^+^ electrolyte that has garnered attention is the Na Superionic Conductor (NASICON), Na_1+*x*_Zr_2_Si*_x_*P_3−*x*_O_12_ (0 ≤ *x* ≤ 3) [21]. As its name suggests, phase-pure NASICON exhibits high Na^+^ conductivities on the order of 10^−3^ S cm^−1^ at 25°C, the same order of magnitude conductivity as β-alumina Na^+^ electrolytes [21]. However, this electrolyte exhibits instability to molten sodium salts, limiting battery applications. Additionally, ionically insulating ZrO_2_ impurities lower conductivity values. When contemplating other metals for energy storage applications, Mg^2+^ conduction presents exciting opportunities as well as unique challenges due to its highly polarizing nature. Mg^2+^ ion solid electrolytes include Mg(BH_4_)_2_ and MgZr_4_(PO_4_)_6_, which feature relatively modest conductivities of 10^−9^ to 10^−7^ S cm^−1^, respectively, even at greater than 100°C. Notably, the best Mg^2+^ ion solid conductor is in fact a MOF, Mg_2_(dobpdc), impregnated with Mg(OPhCF_3_)_2_ (^−^OPhCF_3_ = 4-trifluoromethylphenolate) and Mg(TFSI)_2_ (TFSI^−^ = bis(trifluoromethanesulfonyl)imide) [22]. This material features a Mg^2+^ conductivity of 10^−4^ S cm^−1^ at 25°C and will be discussed in greater detail below.

**Figure 1.**
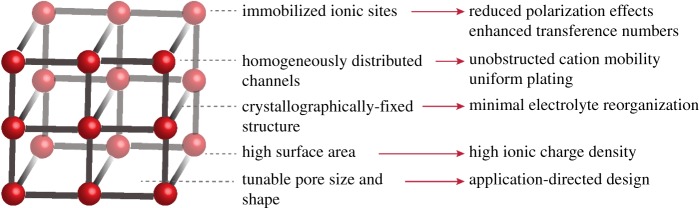
Attractive features of MOFs and COFs as solid-state electrolytes. (Online version in colour.)

Demonstrating the highest Mg^2+^ ion conductivity among solids notwithstanding, MOFs and COFs possess an arsenal of additional properties that identify them as attractive candidates for solid-state electrolytes [23–27] (figure 1). Firstly, the high surface area of MOFs and COFs, which is commonly thousands of m^2^ g^−1^ [28], enables a high density of metal cations and hopping sites, contributing to a maximized power density in a compact device. The long-range order and well-defined ion conductivity pathways in MOFs and COFs provides affords efficient ion shuttling while reducing much of the diffusion limitations associated with non-porous solids, especially for highly polarized species. The crystallographic definition offers homogeneously dispersed hopping sites while eliminating impedance stemming from electrolyte reorganization, as seen with liquid and polymer electrolytes [13,16]. The electronic structure of MOFs and COFs is also beneficial in that their composition rarely offers a high density of mobile electrons or holes, with most materials in this class being excellent electrical insulators [29]. This insulating character is an essential property of the electrolyte, so as to separate the anode and cathode and prevent short circuiting. Porous solid-state electrolytes can also aid in optimizing cation transference numbers; liquid electrolytes often exhibit cation transference numbers of less than 0.4 because both the cations and anions are mobile and thus both contribute to current passed [30,31]. Conversely, anions can be coordinated to or integrated directly into the MOF/COF structure and are therefore immobilized during battery charging and discharging, enhancing battery efficiency. Not only can such materials be used to immobilize anions, but they can also trap by-products that may be generated during battery cycling that otherwise decrease battery lifetime upon contact with the electrodes [32]. Further, because pores can host liquid electrolytes without leakage, porous solids offer the dielectric benefits of liquid electrolytes without the safety concerns of the latter. Finally, synthetic tunability of MOFs and COFs is a powerful feature: the ability to alter the pore size, polarity, material density, metal (in the case of MOFs) and anion identity, as well as the coordination environment enables the design of a host of electrolytes featuring a wide range of properties that can meet a variety of device-specific criteria.

Although beyond the scope of this review, it should be noted that the tunability of MOFs and COFs in terms of their p*K*_a_ and water stability makes them effective proton conductive electrolytes for proton exchange membranes [33–36]. Indeed, most studies of ion conduction in these materials have focused on proton conduction, but emerging in the past decade have been pioneering investigations of Groups 1 and 2 metal ion conduction. Whereas proton conductivity often relies on the installation of acidic functional groups within the framework, conduction of metal ions has different requirements, as will be discussed below.

## 3. Metal ion conduction in MOF/COF composites

The utility of MOFs/COFs as solid electrolytes is highlighted both by their intrinsic properties and by their role in composites with polymers and ionic liquids (ILs). As part of composites, the ordered, crystalline nature of these materials aids in controlling polymer and IL aggregation by housing the polymers or ILs within the pores, while still retaining the ionically conductive and non-flammable properties of the polymers and ILs themselves. In polymer composites, materials such as polyethylene oxide (PEO) or polyethylene glycol (PEG) are commonly incorporated into the evacuated MOF/COF pores by stirring the latter in organic solutions of Li-containing polymers or by a solvent-free, hot press method. Access to an arsenal of composite formulation techniques allows for tailoring the electrolyte preparation procedure to accommodate limitations of a given host, e.g. mechanical instability, incompatibility to certain solvents, etc. Such polymer composites exhibit ionic conductivity values between 10^−6^ and 10^−4^ S cm^−1^ [37–42], higher by up to two orders of magnitude compared to polymer-Li salt composites alone [41]. Co-formulation of MOFs and COFs with polymers has been thoroughly reviewed previously [23]. Although entrapping the polymers within the host pores can prevent polymer crystallization and aggregation, which in turn enhances conductivity, this approach to electrolyte development did introduce several challenges. Filling the pores with a guest material significantly decreases surface area, which in turn contributes to higher diffusion limitations for ion migration, effectively nullifying one of the inherent advantages of porous materials as solid electrolytes. Additionally, the reported alkali metal transference numbers for these electrolytes are never higher than 0.55, and can be as low as 0.34, offering little to no improvement over liquid electrolytes [38–40,42]. These modest transference numbers indicate that although encaging the polymers within the MOFs or COFs does enhance conductivity, this approach fails to immobilize charge-balancing anions and other mobile species. Finally, the challenge with predicting the ultimate properties of the composites, or understanding their interfacial structure, makes rational design of such electrolytes difficult.

Incorporation of ILs into MOFs and COFs pores has also produced composites with some notable properties as solid electrolytes. Isolating ILs within confined micropores is particularly desirable because it can change the phase transition temperature of certain ILs that otherwise solidify and are therefore not usable at ambient temperature [43]. The four primary strategies for impregnating MOFs and COFs with ILs are soaking the material in an IL with or without a co-solvent, allowing the IL to anchor to coordinatively unsaturated sites within the porous host; the ‘ship in a bottle’ method wherein precursors for the IL are introduced inside of the MOF/COF such that the final IL assembles within the pores; capillary action-promoted diffusion of the IL into the pores [43,44]; and one-pot assembly of the IL composite [45,46]. An appropriate method may be chosen based on the presence or absence of coordinatively unsaturated sites in the MOF/COF, the sizes of the aperture openings, and the molecular sizes of the ILs themselves.

An early example of a MOF-IL composite as a solid electrolyte was reported by Fujie, Kitagawa and co-workers [47], who physically blended Zn(2-methylimidazole)_2_ (ZIF-8) with EMI-TFSI (1-ethyl-3-methylimidazolium trifluoromethanesulfonimide)/LiTFSI to obtain an electrolyte. A low activation energy of 0.16 eV and an ionic conductivity of 10^−5^ S cm^−1^ at 25°C was reported for this composite, which nevertheless was lower than that of the MOF-IL combination alone, measured in the absence of the Li salt. Blending EMI-TFSI/Li-TFSI with Zr_6_O_4_(OH)_4_(H_2_TCPP)_3_ (MOF-525, H_2_TCPP = tetracarboxyphenylporphyrin) gave an electrolyte with a conductivity of 10^−4^ S cm^−1^ and a Li^+^ transference number of 0.36 [48]. Although still only in the same range as liquid electrolytes, the transference number for the MOF-IL composite was a marked improvement upon the transference number measured for EMI-TFSI/LiTFSI itself, and was attributed to confinement of the EMI^+^ and TFSI^−^ ions within the MOF pores. These early studies of MOF/ILs composites highlighted certain potential benefits of confining the ILs to micropores, but also revealed unexpected results such as diminished conductivity upon addition of Li^+^. A similar trend was observed in a composite of ZnO_4_(BDC)_3_ (MOF-5, BDC^2−^ = 1,4-benzenedicarboxylate) with AMImTFSI (1-allyl-3-methylimidazolium TFSI) [45]. Doping this composite with increasing amounts of LiTFSI afforded electrolytes with gel-like consistencies with good ionic conductivities of 10^−3^–10^−2^ S cm^−1^ at 51°C, which showed inverse dependence with the amount of Li^+^. Although the authors attributed this unexpected observation to a change to a more tortuous Li^+^ conduction pathway in the more highly loaded samples, experiments to substantiate such mechanistic implications are difficult and often not pursued in the MOF/COF literature thus far. Regardless, these rather complex composites exhibit impressively low activation energies of less than 0.1 eV and working potential windows greater than 5.2 V, warranting additional future studies. The wide potential windows of the above MOF composites highlight the resilience against reduction or oxidation that solid electrolytes may feature even if the structures contain metal ions.

One word of caution is that both the anion and the cation in an IL have non-zero mobilities within the framework, and both can contribute to overall ionic conductivity, such that the metal cations are not the only charged mobile species within these electrolytes [49]. Measuring the Li^+^ transference numbers of the composites is an important step in identifying the Li^+^ contribution to the conductivity. Additionally, as with polymer composite electrolytes discussed above, understanding the interfacial interaction between the MOF/COF and the IL is difficult, making the discovery of new IL-based composites squarely an empirical challenge with little hope of rational design [46].

## 4. Metal ion conduction in neat MOFs and COFs

### (a) Coordinating anions to open metal or other cationic sites

**Figure 2.**
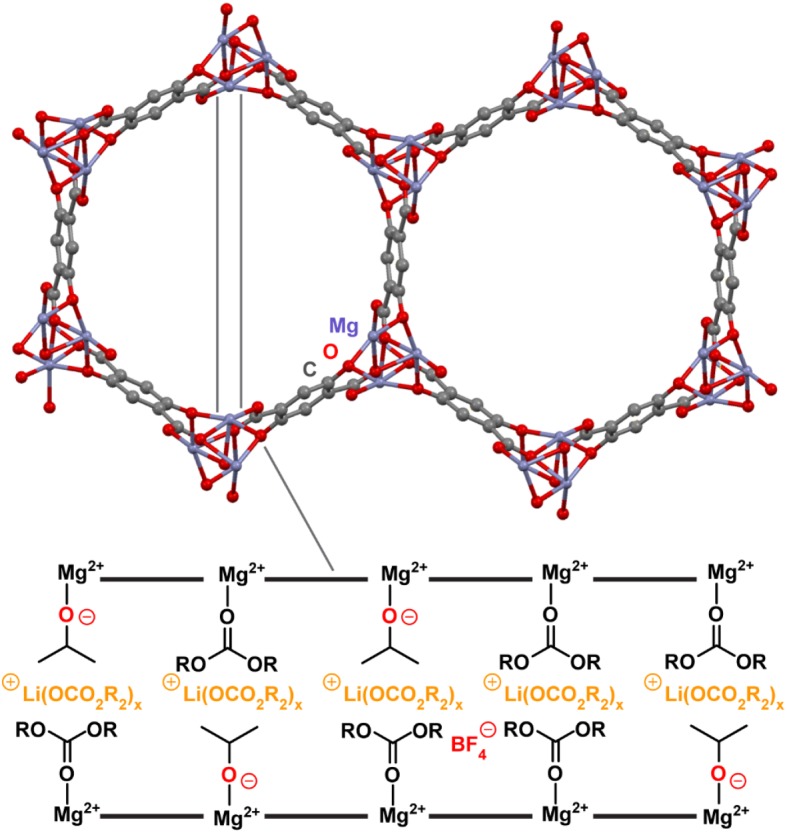
A portion of the structure of Mg_2_(dobdc)·0.35Li*^i^*OPr·0.25LiBF_4_·EC·DEC. The cross-sectional view depicts the envisioned migration path for Li^+^ ions through the electrolyte. H atoms are omitted for clarity.

The structural and compositional tunability of MOFs and COFs is one of the attributes that encourages their exploration as neat solid electrolytes. Although the ability of these materials to intercalate ions has resulted in numerous works detailing their use as battery electrode materials [24,27,50–53], employing them as solid-state electrolytes has emerged only recently. One of the pioneering studies in this context was published in 2011 by Wiers, Long and co-workers [54]. This study reported soaking Zn_4_O(BTB)_2_ (MOF-177, BTB^3−^ = 1,3,5-benzenetribenzoate), H_3_[(Cu_4_Cl)_3_(BTTri)]_8_ (Cu-BTTri, BTTri^3−^ = 1,3,5-tris(1*H*-1,2,3-triazol-5-yl)benzene), and Mg_2_(dobdc) (dobdc^4−^ = 5-dioxido-1,4-benzene-dicarboxylate) in 1 : 1 ethylene carbonate:diethyl carbonate solutions of LiBF_4_ and conducting electrical impedance spectroscopy (EIS) on the pressed pellet samples. The Li^+^-doped MOFs yielded ionic conductivities ranging from 10^−9^ to 10^−6^ S cm^−1^, with the most promising host being Mg_2_(dobdc). Although an intriguing early result, the ionic conductivity of 1.8 × 10^−6^ S cm^−1^ observed in Mg_2_(dobdc) was still at least two orders of magnitude lower than the technological benchmark for battery applications [13]. Taking advantage of the coordinatively unsaturated Mg^2+^ sites in this framework, the authors added Li*^i^*OPr and showed that coordination of ^‒*i*^OPr to these sites immobilized the anions and allowed the cations to move more freely, further increasing the conductivity by a factor of 10. The optimized electrolyte, Mg_2_(dobdc)·0.35Li*^i^*OPr·0.25LiBF_4_·EC·DEC (EC = ethylene carbonate, DEC = diethyl carbonate) (figure 2) exhibited a conductivity of 3.1 × 10^−4^ S cm^−1^ and an activation energy of 0.15 eV, meeting superionic conductor qualifications [55]. The need for LiBF_4_ in this optimized formulation was justified by implicating it in inter-particle conductivity, with EC and DEC solvating the Li^+^ ions in the pores and improving inter-particle contacts.

**Figure 3.**
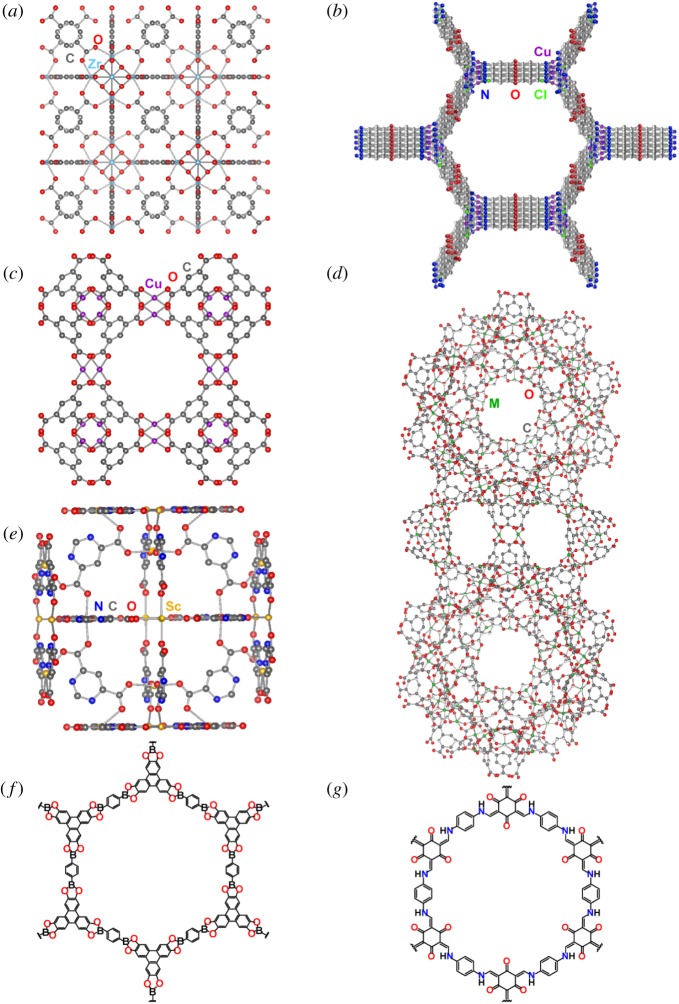
Structures of MOFs and COFs that have served as pioneers in the porous solid electrolyte field: (*a*) UiO-66 (isomorphic with UiO-67), (*b*) MIT-20, (*c*) HKUST-1, (*d*) MIL-100 (M = Cr^3+^ Fe^3+^, or Al^3+^), (*e*) [Sc*X*(μ_4_-pmdc)_2_(H_2_O)_2_]·5H_2_O, (*f*) COF-5, and (*g*) TpPa-1. Water molecules and H atoms are omitted. (Online version in colour.)

The Long group later expanded upon the notion of immobilizing charge-balancing anions on open metal sites in MOFs in order to maximize exclusively Li^+^ mobility. The framework UiO-66 (Zr_6_O_4_(OH)_4_(BDC)_6_) can undergo thermal dehydration to afford coordinatively-unsaturated Zr^4+^ sites [56,57] (figure 3*a*). Ameloot, Long and co-workers capitalized on this feature by soaking the dehydrated UiO-66 in a tetrahydrofuran solution of Li-O*^t^*Bu, consequently saturating the Zr^4+^ coordination sphere with alkoxide anions and incorporating charge-balancing Li^+^ cations [58]. The resulting Li^+^ ionic conductivity was reported to be 1.8 × 10^−5^ S cm^−1^, one order of magnitude lower than the reported Mg_2_(dobdc)·0.35Li*^i^*OPr·0.25LiBF_4_·EC·DEC [54] but still competitive with solid polymer electrolytes [14,59]. Further, the bulky aliphatic groups on the alkoxide shield the negative charge of the anion, thus weakening the interaction between the anion and the Li^+^ cations and enabling a low Li^+^ activation energy of 0.18 eV. Unfortunately, a symmetric Li cell with this electrolyte could only be cycled three times before shorting due to Li dendrite formation. It may be possible that altering the pore shapes/channel orientations may allow better control over the uniformity of Li plating, which could aid in decreasing dendrite formation. If dendrites formed along grain boundaries, forming larger host crystals and thus decreasing grain boundary density, or adding a polymeric binder to mitigate the effects of grain boundaries, may also help eliminate dendrite formation.

In addition to Li^+^ conduction, another promising application of porous material-based electrolytes is conduction of more charge-dense Mg^2+^ ions. One consideration when designing materials for Mg^2+^ conduction is the larger size of Mg^2+^ ions compared to that of Li^+^, particularly when solvated, which necessitates larger pore size to allow Mg^2+^ transport. Aubrey, Long and co-workers explored both Mg_2_(dobdc) (structure shown in figure 2) and its expanded analogue Mg_2_(dobpdc) (dobpdc^4−^ = 4,4′-dioxidobiphenyl-3,3′-dicarboxylate) as Mg^2+^ ion conductors [22]. In line with the expanded pore size of Mg_2_(dobpdc) compared to that of Mg_2_(dobdc) (diameter = 21 Å versus 13 Å), Mg_2_(dobpdc) could accommodate more than three times the mole equivalents of free Mg^2+^ ions than the Mg_2_(dobdc) host and more than two times the mole equivalents of the dielectric triglyme, which was added to all samples. This is accompanied by a more than 100-fold increase in conductivity, with conductivity values of approximately 10^−4^ S cm^−1^ observed in Mg_2_(dobpdc)-Mg(TFSI)_2_ and approximately 10^−6^ S cm^−1^ observed in Mg_2_(dobdc)-Mg(TFSI)_2_. Champion devices made from soaking the MOFs in Mg(TFSI)_2_ and Mg(OPhCF_3_)_2_ afforded conductivities of 10^−4^ S cm^−1^ with Mg_2_(dobdc) and slightly higher with Mg_2_(dobpdc). These conductivity values are higher than those reported for any solid Mg^2+^ electrolytes and, combined with low activation energies of 0.11–0.19 eV, render the materials relevant for commercial applications. Studies of the stability of these materials to prolonged cycling and to Mg metal or other electrode materials would be useful for exploring the potential of these MOFs in a battery assembly.

The ability to coordinate a variety of anions to open metal sites in MOFs and impart conductivity of various cations introduces the opportunity to establish material-specific trends in performance. For example, Park, Tulchinsky and Dincă reported an anionic Cu^II^-azolate MOF, (CH_3_)_2_NH_2_ [Cu_2_Cl_3_BTDD]·(DMF)_4_(H_2_O)_4.5_ (MIT-20, H_2_BTDD = bis(1H-1,2,3-triazolo[4,5-*b*],[4′,5′-*i*])dibenzo-[1,4]dioxin) that featured charge-balancing dimethylammonium cations (figure 3*b*) [60]. Presence of free dimethylammonium cations in the parent structure suggested that the MOF could accommodate and potentially conduct metal cations. Removal of residual DMF and water molecules, as well as one equivalent of dimethylammonium chloride afforded a neutral framework, Cu_2_Cl_2_BTDD. The thermodynamic favouring of the anionic framework during synthesis allowed a quantitative yield of the anionic MIT-20 charge-balanced by free Group 1 and 2 metal cations when soaking in the respective metal salts. Because this quantitative transformation of MIT-20 affords isostructural materials regardless of the nature of the anion, soaking the MOF in LiCl, LiBr, and LiBF_4_ salts with addition of the dielectric propylene carbonate (PC) enabled the exploration of the effect of anion identity on electrolyte performance. Gratifyingly, an increasing softness of the anion correlated well with increasing Li^+^ conductivity (10^−5^ S cm^−1^ to 10^−4^ S cm^−1^) and decreasing activation energy (0.32–0.16 eV). A Li^+^ transference number of 0.66 was measured for MIT-20-LiCl, confirming that the primary contributor to the conductivity was mobile Li^+^, and the Cl^−^ anions were conversely immobilized on the open metal sites in the framework. MIT-20 also exhibited good Na^+^ and Mg^2+^ conductivity (*σ*_Na_ = 1.8 × 10^−5^ S cm^−1^ and *σ*_Mg_ = 8.8 × 10^−7^ S cm^−1^) and activation energies of 0.39 eV and 0.37 eV upon soaking in solutions of NaSCN and MgBr_2_, respectively. The ability to install both different anions and different cations within the MIT-20 structure highlights the versatility of this material as a solid electrolyte. This material also exemplifies the generalization that MOFs and COFs which have isostructural phases that are isolable in multiple states of formal charge could be promising candidates for ionically conductive solid electrolytes.

Another example of capitalizing on the modularity of MOFs to establish structure–function relationships was Shen, Dunn and co-workers' exploration of the MIL-100 and UiO series of MOFs as tunable solid electrolytes [61]. A proof-of-principle was demonstrated by targeting the installation of $\text{Cl}{{\text{O}}_{\text{4}}}^{-}$ ions from a PC solution of LiClO_4_ onto the coordinatively unsaturated Cu^2+^ sites of activated Cu_3_(BTC)_2_ (HKUST-1, BTC^3−^ = benzene-1,3,5-tricarboxylate) (figure 3*c*), allowing Li^+^ ions to move freely upon polarization for a Li^+^ conductivity of 3.8 × 10^−4^ S cm^−1^ and an activation energy of 0.18 eV. A similar PC-LiClO_4_ treatment of materials in the activated MIL-100 series (M_3_O(BTC)_2_OH, M = Cr^3+^ Fe^3+^, or Al^3+^) (figure 3*d*) produced solids whose conductivity ranged from 10^−3^ S cm^−1^ to 10^−2^ S cm^−1^. The highest ionic conductivity of the MIL-100 MOFs, observed with MIL-100-Al^3+^, was consistent with the assertion that the increased Lewis acidity of Al^3+^ compared to Fe^3+^ and Cr^3+^ led to decreased ion pairing strength between the $\text{Cl}{{\text{O}}_{\text{4}}}^{-}$ and the Li^+^, thus enhancing Li^+^ mobility. The effect of MOF pore size on ionic conductivity was also explored using activated UiO-66 and the larger-pore Zr_6_O_4_(OH)_4_(BPDC)_6_ (UiO-67, BPDC^2−^ = biphenyl-4,4′-dicarboxylate). Soaking these MOFs in PC solutions of LiClO_4_ gave Li^+^ conductivities of 1.8 × 10^−4^ S cm^−1^ and 6.5 × 10^−4^ S cm^−1^ for UiO-66 and UiO-67, respectively. The higher Li^+^ conductivity observed in UiO-67 was attributed to the larger pore size being able to accommodate a higher extent of solvation around the Li^+^ ions, which enhances mobility. This trend was consistent with that observed in Mg_2_(dobdc) versus its expanded analogue Mg_2_(dobpdc), as discussed earlier. Lower activation energy was measured in UiO-67 versus UiO-66 as well (*E*_a_ = 0.12 eV versus 0.21 eV for UiO-67 versus UiO-66, respectively).

In addition to coordinating anions to open metal sites as cation hopping sites within MOFs, anions have also been incorporated into positively charged MOF/COF structures simply through weaker Coulombic interactions. Recently, Chen and co-workers reported a cationic COF comprising alternatively linked triaminoguanidinium and 1,3,5-triformylphloroglucinol ligands which was proposed to feature π−π stacking, forming channels from the aligned pores [62]. Stirring the COF in an aqueous solution of LiTFSI replaced the parent chloride ions with TFSI^−^ ions. One equivalent of TFSI^−^ was charge-balancing the triaminoguanidinium within the framework and one equivalent was charge-balancing Li^+^ ions which remained in the electrolyte matrix. This electrolyte exhibited a conductivity of 5.74 × 10^−5^ S cm^−1^ at 30°C and an activation energy of 0.34 eV. The Li^+^ transference number of 0.61 was consistent with at least a portion of the TFSI^−^ ions being immobilized through interaction with the cationic triaminoguanidinium groups. Additionally, a respectable operating potential window of 3.8 V was measured. Studies suggested that the TFSI^−^ ion existed within the framework both as a ‘free’ anion stabilized within the COF channel through Coulombic interactions, and as an ion pair. The ion-paired TFSI^−^ likely decreases the Li^+^ transference number, given that the equivalent of TFSI^−^ present within the framework to charge-balance the Li^+^ is likely not coordinated to the COF. Although this example showcases post-synthetic alteration of the anion identity that is not feasible in borate-based COFs (see below), the challenge with using a cationic COF rather than a coordinatively unsaturated charge-neutral MOF is that addition of alkali metal salts such as LiTFSI introduces equivalents of monoanions both to charge-balance the cationic framework and to ion-pair with the metal cations. Such electrolytes still possess an advantageously high density of anionic hopping sites and the safety features of solid electrolytes, but obtaining higher metal cation transference numbers will likely be a challenge due to the large percentage of mobile anions. An interesting extension upon this work could involve soaking the COF in a polylithium salt [63–67]. This could yield the COF with equal equivalents of the polyanion immobilized within the channels, triaminoguanidinium groups within the framework itself and mobile Li^+^. One consideration with this approach would be careful selection of the anion, particularly in terms of size; Chen *et al.* reported diminished π−π stacking within the COF upon replacing the Cl^−^ ions with larger TFSI^−^ ions. This partial collapse of the stacked structure may obstruct metal transport pathways within the framework.

### (b) Incorporating anions directly into the structure

An alternative to introducing stoichiometric equivalents of anions concomitant with mobile cations is to target inherently negative frameworks, where the negative charges are built into the MOF/COF building blocks themselves. In 2015, Van Humbeck, Long and co-workers reported cross-linked tetraarylborate moieties that form a negatively charged porous polymer wherein the anionic borates serve as immobile Li^+^ hopping sites [68]. This approach was inspired by early reports of linear polymers containing ionic groups such as anionic perfluoroalkyl carboxylates [69] or cationic diallyldimethylammonium units [70] within the polymer structure. Such polymers were also used as solid electrolytes with the goal of achieving single-ion conductivity, and exhibited mobile ion transference numbers nearing unity. The observed conductivities for such polymers fell in the 10^−6^ to 10^−5^ S cm^−1^ range, possibly due to undesirably large distances between the hopping sites along the polymer backbone. In contrast, the material designed by Van Humbeck, Long *et al*. features an interpenetrated network that provides a high density of ion hopping sites. Measurements gave a Li^+^ conductivity of 3.6 × 10^−5^ S cm^−1^, which increased further by one order of magnitude upon perfluorination of the aryl groups in the tetraarylborate network (*σ*_Li_ = 2.7 × 10^−4^ S cm^−1^). Installation of electron-withdrawing fluorine atoms on the aryl rings was thought to weaken the borate–Li^+^ interaction and thus encourage Li^+^ mobility through the electrolyte. As expected, immobilization of the anions within the framework afforded strong single-ion conducting character with a high Li^+^ transference number (*t*_Li+_ = 0.9). Interestingly, altering synthetic conditions afforded a permanently porous fluorinated tetraarylborate material (BET surface area = 480 m^2^ g^−1^) that exhibited 10-fold lower conductivity than its dense-phase congener. Although this difference in conductivity between the porous and dense phase is consistent with the need for closely packed hopping sites, the activation energies of the two phases were identical, 0.25 eV. The phase-independent activation energy data may point to dominating surface conduction pathways as proposed by the authors. However, the distinct differences in conductivity as a function of phase density and the fact that the identities of the hopping sites do remain constant in both phases highlights the importance of hopping site density on ionic conductivity. Most tellingly, it emphasizes that three-dimensionally connected pores become detrimental to ion transport beyond a certain diameter.

**Figure 4.**
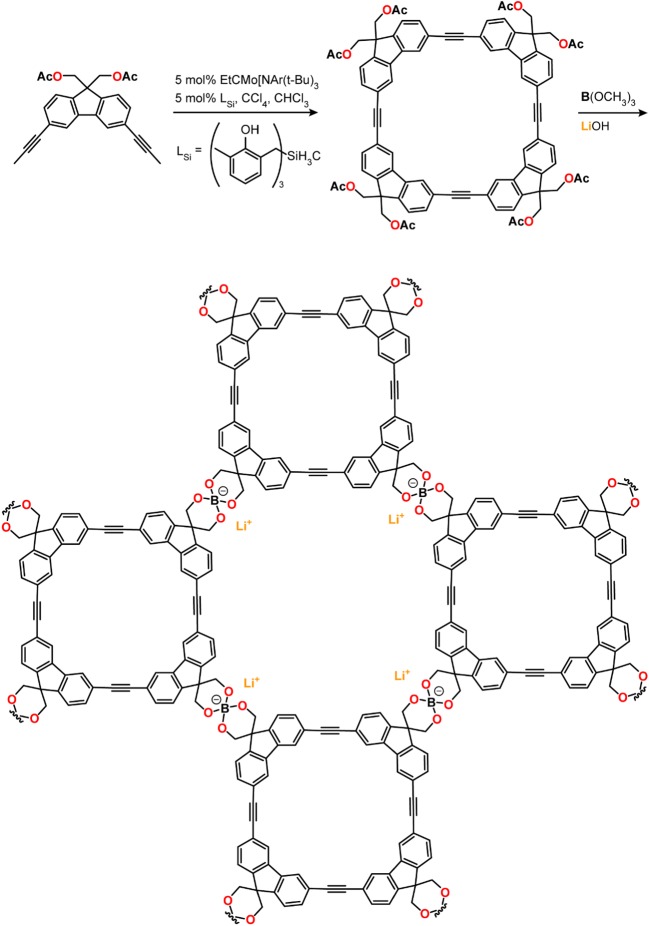
Spiroborate COF featuring anionic sites integrated into structure, and charge-balancing Li^+^ ions provided by the base during synthesis. (Online version in colour.)

Another example of anionic borates being featured in ionically conductive MOFs and COFs was a spiroborate-based COF synthesized by base-promoted transesterification of diols and trimethylborate using LiOH as the base. The latter served the roles of both deprotonating the diol during the transesterification and providing the Li^+^ ions for the electrolyte [71], thus allowing a one-pot synthesis of a Li^+^-loaded solid electrolyte (figure 4). Incorporating the spiroborate structure into the COF was motivated by previous reports of Li borate salts used as Li^+^ electrolytes [72]. The spiroborate COF/polyvinylidene fluoride (PVDF) formulation exhibited a Li^+^ conductivity of 3.05 × 10^−5^ S cm^−1^ and an activation energy of 0.24 eV. In addition to favourable conductivity and activation energy values, a high Li^+^ transference number of 0.8 was measured. Finally, a respectable potential window of *ca* 4.5 V was reported, further highlighting the utility of solid-state electrolytes over liquid electrolytes that decompose at lower potentials. The formation of inherently negatively charged frameworks provides a host matrix with a homogeneous distribution of cation hopping sites that contributes nothing to increasing the anion transference number. It provides a promising blueprint for very efficient cation conductors, but has only rarely been used thus far.

One instance where this strategy proved effective with MOFs involved the substitution of trivalent Sc^3+^ ions in [Sc*X*(μ_4_-pmdc)_2_(H_2_O)_2_]·5H_2_O (pmdc^2−^ = pyrimidine-4,6-dicarboxylate; *X* = Li^+^ or Na^+^) (figure 3*e*) by divalent Cd^2+^ or Mn^2+^ [73]. Low conductivity in the parent samples presumably stems from the alkali metal cations being affixed in the framework, thus hindering their mobility. As such, aliovalent substitution of Sc^3+^ with Cd^2+^ or Mn^2+^ was pursued with the goal of installing additional free alkali metal cations for charge balance. Indeed, free Li^+^ and Na^+^ ions compensated for the charge imbalance created by this aliovalent substitution. However, these ions still contributed low Li^+^ and Na^+^ conductivity values (10^−7^ to 10^−6^ S cm^−1^ for the Cd^2+^ and Mn^2+^-doped MOFs). The authors reported enhanced Li^+^ and Na^+^ conductivity values from simply soaking the parent Sc^3+^-MOFs in solutions of LiBF_4_ or NaPF_6_. The resulting electrolytes exhibited ionic conductivities of 10^−5^ S cm^−1^ (Na^+^) and 10^−4^ S cm^−1^ (Li^+^). Even though the soaking procedure afforded enhanced conductivity, this treatment also caused cracking of the crystals, while peak broadening in the ^1^H and ^7^Li nuclear magnetic resonance (NMR) spectra revealed increased structural heterogeneity. Together, these observations complicate the direct correlation between conductivity and the mobile charge density and prevent detailed studies probing potentially new conduction mechanisms in the metal-exchanged samples. Such information could have been useful in explaining the large discrepancy in activation energies for the Li samples versus the Na samples (0.25 eV versus 0.64 eV, respectively). Despite the low conductivity observed in the aliovalently doped samples described above, the idea that aliovalent substitution can increase the mobile cation density in a MOF is potentially quite general and could in principle be applied to MOFs made from tri- or higher-valent metal ions, with cation substitution in general established as a versatile synthetic technique in this class [74].

### (c) Neutral host frameworks

Although neutral host frameworks that do not easily accommodate anions may not seem like ideal candidates for solid electrolytes, some notable examples that highlight the importance of processing porous solid electrolytes do use such hosts. For instance, uniaxial pressure applied to C_9_H_4_BO_2_ (COF-5) (figure 3*f*) and TpPa-1 (Tp = triformylphloroglucinol, Pa = paraphenylenediamine) (figure 3*g*) promoted preferred orientation of platelet crystallites, thereby forcing alignments of the COF pores and the formation of long-range channels for more efficient ion transport [75]. Soaking these materials in solutions of LiClO_4_ followed by evaporation and uniaxial pressing afforded solid electrolytes with conductivities of 2.6 × 10^−4^ S cm^−1^ and 1.5 × 10^−4^ S cm^−1^ for COF-5 and TpPa-1, respectively. Although ^7^Li NMR experiments confirmed the highly mobile nature of Li^+^, the mobility of the charge-balancing $\text{Cl}{{\text{O}}_{\text{4}}}^{-}$ anions which can also contribute to the conductivity was not measured. As before, obtaining the Li^+^ transference numbers for these COFs would be critical for assessing the Li^+^ contribution to the total ionic conductivity.

Recently, the notion of forming true hybrids between porous materials and polymers for ion conduction has been pursued in the form of the ‘polyelectrolyte’ COFs such as TPB-DMTP-COF (TPB = 1,3,5-tri(4-aminophenyl)benzene, DMTP = 2,5-dimethoxyterephthalaldehyde) and TPB-BMTP-COF (BMTP = 2,5-bis((2-methoxyethoxy)methoxy)terephthalaldehyde) [76]. Condensation of TPB with either DMTP or BMTP resulted in porous, stacked two-dimensional COFs with either methoxy groups (TPB-DMTP-COF) or oligo(ethylene oxide) chains (TPB-BMTP-COF) branching off of the phenyl rings and lining the pore walls. This approach aimed to combine the ion transport benefits of polymer electrolytes with the mechanical and thermal stability of MOF/COF electrolytes. Soaking these porous, crystalline structures in solutions of LiClO_4_ afforded materials with conductivities of 10^−7^ S cm^−1^ (TPB-DMTP-COF) and 10^−6^ S cm^−1^ (TPB-BMTP-COF) at 40°C, both improvements upon that of the PEO-Li^+^ complex, which has a Li^+^ conductivity of 10^−8^ S cm^−1^ at 40°C. It should be noted that both materials exhibit high activation energies for ionic transport, 0.96 eV for TPB-DMTP-COF and 0.87 eV for TPB-BMTP-COF, which suggests that improvements are likely for a class of materials that allows for considerable combinatorial potential. Once again, more systematic improvements would be facilitated by ^7^Li NMR studies and measurements of the Li^+^ transference numbers to parse out the mobility of free Li^+^ ions versus ion-paired LiClO_4_. The approach of implementing polymeric building blocks that have proven ion conductivity into crystallographically well-defined and mechanically and thermally robust COF structures is intriguing. Further structural characterization of these analogues after incorporation of Li salts would aid in determining whether the COF structure is retained in the final electrolyte matrix.

## 5. Scouting criteria: what makes a MOF/COF ionically conductive?

The several examples discussed herein were chosen to showcase the multiple approaches available for achieving ionic conductivity in MOFs and COFs. When evaluating these porous materials as potential candidates for ion conduction, the following considerations may prove useful:

— Does the MOF feature metal sites with coordination environments that include removable solvent molecules, or other anion docking sites?
— Are anions incorporated into the MOF/COF structure, e.g. as part of the building blocks or by Coulombic forces?
— Is the material isolable in multiple states of formal charge? Is isolation of these states reversible?
— Is the MOF/COF electrically insulating?
— Is there a high density of hopping sites within the structure?
— What are the sizes of metal ions that could be accommodated within the pores?
— Would the mobile metal ions be solvated in the pores? How many equivalents of solvated metal ions can the structure accommodate?

When testing MOFs and COFs for ion conduction, the following criteria can serve as reference benchmarks for evaluating performance:

— Ionic conductivity ≥10^−4^ S cm^−1^, ideally when *T *∼ 25°C [55]
— Electrical conductivity ≤10^−10^ S cm^−1^, to avoid cell shorting [13]
— Activation energy ≤0.4 eV [55]
— Working potential window of ≥4 V for commercial applications [77]
— Transference number of ≥0.5, to avoid polarization effects [60]
— Structurally stable to the desired mobile metal salts, dielectric additives, and the electrode materials
— No or nominal increase in resistance during cycling

## 6. Conclusions/future directions

The continuously growing energy demand is being addressed with innovative, sustainable technology and metal and metal-ion batteries remain leaders for electrical energy storage in terms of combined energy density, portability, longevity and cost. Continued optimization of these devices requires enhanced safety and even greater operating efficiency, both of which can be greatly improved by an optimized solid-state electrolyte. MOFs and COFs have gained attention as promising candidates for solid-state electrolyte technology due to their crystallographic definition which contributes immobilized and homogeneously distributed ion hopping paths, enhanced thermal and mechanical stability, and a morphology that in principle could prevent hazardous dendrite formation. The high surface area of these materials allows an abundance of cation hopping sites, which aids in minimizing battery resistance. Reports detailing installation of hopping sites into MOFs and COFs both by coordinating anions to cationic sites within the frameworks and by installing anionic sites directly as components of the frameworks highlight the versatility of this class of materials for battery electrolyte applications. Several examples have shown great promise in this arena by exhibiting ionic conductivities of 10^−6^ to 10^−4^ S cm^−1^ under ambient conditions, activation energies of 0.1–0.4 eV, cation transference numbers of 0.6–0.9, and potential windows exceeding 4.0 V.

Looking forward, exploring the effect of MOF/COF crystal size on conductivity could aid in elucidating whether ion mobility is an inter- or intra-crystal phenomenon. Such studies could also aid in optimizing conductivity versus dendrite formation, which may occur along grain boundaries. Further, many of the reported MOFs and COFs have shown promising properties when combined with monolithium salts. Expansion of these studies could involve use of a polylithium salt, to achieve higher Li^+^ loading. Another underexplored area is the utilization of inherently anionic materials balanced by potentially mobile cations residing in the pores. This may encourage homogeneous distribution of the charge balancing metal ions throughout the host matrix while minimizing incorporation of mobile, exogenous species that are typically introduced using more iterative electrolyte preparation methods. Additionally, hybridizing porous materials with traditional polymer electrolytes may allow for retention of the ionically conductive properties of the polymers while adding benefits associated with porous solid electrolytes, e.g. minimized electrolyte reorganization, maximized hopping site density, and potentially no dendrite growth. Finally, targeting good ion conductors for K^+^, Ca^2+^ or Al^3+^ transport in the MOF/COF context could prove fruitful given that these larger/higher-valent ions might require larger pores than typically available with denser materials. Employing MOFs and COFs as solid electrolytes for K-ion, Ca-ion or Al-ion batteries would combine the benefits of porous material-based electrolytes with the advantages of using energy-dense, earth-abundant ions. The wealth of metal and ligand combinations that may engender a host of pore shapes, sizes, and local electronic environments that may accommodate any number of metal ions lays an expansive foundation for a bright future of MOF/COF-based solid electrolytes.

## Data accessibility

This article has no additional data.
